# Simultaneous selection for grain yield and protein content in genomics-assisted wheat breeding

**DOI:** 10.1007/s00122-019-03312-5

**Published:** 2019-02-27

**Authors:** Sebastian Michel, Franziska Löschenberger, Christian Ametz, Bernadette Pachler, Ellen Sparry, Hermann Bürstmayr

**Affiliations:** 1 Department of Agrobiotechnology, IFA-Tulln, University of Natural Resources and Life Sciences Vienna, Konrad-Lorenz-Str. 20, 3430 Tulln, Austria; 2 Saatzucht Donau GesmbH & CoKG, Saatzuchtstrasse 11, 2301 Probstdorf, Austria; 3 C&M Seeds, 6180 5th Line, Palmerston, ON N0G 2P0 Canada

## Abstract

**Key message:**

Large genetic improvement can be achieved by simultaneous genomic selection for grain yield and protein content when combining different breeding strategies in the form of selection indices.

**Abstract:**

Genomic selection has been implemented in many national and international breeding programmes in recent years. Numerous studies have shown the potential of this new breeding tool; few have, however, taken the simultaneous selection for multiple traits into account that is though common practice in breeding programmes. The simultaneous improvement in grain yield and protein content is thereby a major challenge in wheat breeding due to a severe negative trade-off. Accordingly, the potential and limits of multi-trait selection for this particular trait complex utilizing the vast phenotypic and genomic data collected in an applied wheat breeding programme were investigated in this study. Two breeding strategies based on various genomic-selection indices were compared, which (1) aimed to select high-protein genotypes with acceptable yield potential and (2) develop high-yielding varieties, while maintaining protein content. The prediction accuracy of preliminary yield trials could be strongly improved when combining phenotypic and genomic information in a genomics-assisted selection approach, which surpassed both genomics-based and classical phenotypic selection methods both for single trait predictions and in genomic index selection across years. The employed genomic selection indices mitigated furthermore the negative trade-off between grain yield and protein content leading to a substantial selection response for protein yield, i.e. total seed nitrogen content, which suggested that it is feasible to develop varieties that combine a superior yield potential with comparably high protein content, thus utilizing available nitrogen resources more efficiently.

**Electronic supplementary material:**

The online version of this article (10.1007/s00122-019-03312-5) contains supplementary material, which is available to authorized users.

## Introduction

The implementation of genomic selection in many national and international plant breeding programmes in recent years (Guzmán et al. 2016; Lado et al. 2016; Michel et al. 2016; Cericola et al. 2017; Belamkar et al. 2018; Juliana 2018) highlights the potential of this new breeding tool for variety development and accelerating the genetic improvement in crop plants. The merit of employing genomic predictions has been frequently tested by cross-validation, but also across families and years taking genomic relationship and genotype-by-environment interaction into account (Gezan et al. 2017; Ben Hassen et al. 2018; Kristensen et al. 2018; Huang et al. 2018; Pembleton et al. 2018). These factors are highly relevant to enable an adequate comparison with phenotypic selection in conventional breeding schemes (Sallam and Smith 2016; Song et al. 2017; Belamkar et al. 2018)and optimizing resource allocations in hybrid and line variety breeding programmes (Longin et al. 2015; Marulanda et al. 2016). Various prediction model extensions have furthermore been proposed for such genomic breeding approaches including the modelling of marker-by-environment interaction (Schulz-Streeck et al. 2013; Bernal-Vasquez et al. 2017;Pérez-Rodríguez et al. 2017), usage of prior information about genotype performance, e.g. from preliminary yield trials (Endelman et al. 2014; Michel et al. 2017) or the inclusion of non-additive effects (Philipp et al. 2016; Akdemir et al. 2017; Jiang et al. 2017, 2018). Furthermore, multi-trait prediction models have been recommended for cases in which prior information from a correlated trait is earlier available or easier to obtain than the main trait of interest (Jia and Jannink 2012; Fernandes et al. 2017; Hayes et al. 2017; Schulthess et al. 2018).

The idea of conducting a simultaneous selection for several traits of interest readily suggests itself for the latter class of prediction models, while converting the specific goals of a breeding programme as well as the desired response to selection for each trait into selection indices aims to maximize the net merit and might significantly ease selection decisions (Bauer and Léon 2008; Schulthess et al. 2016). Such methods can thus be useful to conduct a simultaneous selection for major agronomic traits like grain yield and protein content which poses a major challenge in breeding wheat due to frequently observed strong negative correlation between both traits (Simmonds 1995). This trade-off renders the simultaneous improvement in both traits complicated, and given that the protein content is one important determinant of baking quality (Gabriel et al. 2017) breeders rather aimed to shift this undesirable correlation by increasing grain yield while maintaining the protein content in the past (DePauw et al. 2007). The employment of an index to support selection decisions aims consequently to translate the underlying mechanics of such an approach into a more accessible quantity. The necessary economic weights are however difficult to derive and can, e.g., be set according to the pricing for the involved traits (Haile et al. 2018) or to values that seem to fit the current breeding goals of a given programme (Heffner et al. 2011).

The protein yield has been suggested as one promising alternative selection criterion that specifically targets the protein content/grain yield trade-off as it is equivalent to the total seed nitrogen yield, which has also been systematically improved by breeding in the last decades (McNeal 1982; Simmonds 1995; Acreche and Slafer 2009; Cormier et al. 2013). Another related aspect is the observation that some genotypes deviate from the negative trend seen between grain yield and protein content, which represent thus highly appreciated outliers that possess a comparably higher protein content as would be expected by their respective grain yield. The residuals from this linear regression of protein content on grain yield line have become widely known as grain protein deviation (GPD) (Monaghan et al. 2001) that have been generalized in the regression–residual method proposed by Hänsel (2001) and can be seen as a method to derive yield-adjusted protein content estimates. These adjusted phenotypic breeding values have already shown some potential for mitigating the above-mentioned negative trade-off when used in a recurrent selection scheme (Arief et al. 2010). For this purpose, the grain protein deviations can be calculated on a plot basis by either regressing non-adjusted plot values of protein content on grain yield and deriving the residuals (Monaghan et al. 2001) or firstly fitting a bivariate model for additionally taking design effects into account when estimating the necessary regression coefficients before conducting a multi-environment analysis (Rapp et al. 2018; Thorwarth et al. 2018). The simplest method is though given by calculating the residuals from the regression of protein content on grain yield based on environmental means (Oury and Godin 2007).

Although using at least some of these concepts is common practice in many wheat breeding programmes to conduct a simultaneous selection of grain yield and protein content, they have not yet been tested in combination with various prediction model extensions and in the presence of genotype-by-environment interactions across multiple years that breeders have to face both in conventional and genomic wheat breeding. The aims of this study were thus (i) to compare different concepts for achieving a simultaneous response to selection for grain yield and protein content and (ii) investigate the potential of these concepts in the scope of genotype-by-environment interaction by a forward prediction across multiple years in a applied wheat breeding programme.

## Materials and methods

### Plant material and phenotypic data

A population of 1114 F_4:6_ generation and double haploid winter wheat breeding lines (*Triticum aestivum* L.) derived from more than 600 crosses containing 1–17 individuals per family was analysed in this study. All lines were developed in an applied breeding programme and tested in multi-environment trials under Central and Eastern European conditions from 2010 to 2016. Grain yield (dt ha^−1^), protein content (%), and protein yield (dt ha^−1^) were assessed in unbalanced series of 4–24 multi-environment trials each year depending on the trait. The according phenotypic information from 590 of these lines coming from preliminary yield trials 2012–2015 was additionally included in the analysis and was mostly comprised of unreplicated performance assessments in one location, and year before, multi-environment trials were conducted. Notwithstanding, replicated check varieties were tested along with unreplicated earlier generation lines in preliminary yield trials allowing to correct for spatial field trends according to standard procedure in plant breeding.

### Statistical analysis of phenotypic data

The phenotypic data were analysed in two stages, where each individual yield trial was firstly analysed with various models correcting for row and/or column effects as well as autoregressive variance–covariance structures of the residuals (Burgueño et al. 2000). The best fitting model was chosen by Akaike´s information criterion (AIC) and used to calculate best linear unbiased estimates (BLUE) as well as the repeatability by $h^{2} = \sigma_{\text{G}}^{2} /(\sigma_{\text{G}}^{2} + \frac{1}{2}{\text{MVD}})$, where $\sigma_{\text{G}}^{2}$ designates the genetic variance and ${\text{MVD}}$ the mean variance of a difference of the BLUEs (Piepho and Möhring 2007). Trials with a heritability larger than 0.3 were forwarded to the second stage, where an across trial analysis was conducted using a linear mixed model of the form:

1 $$y_{ij} = \mu + g_{i} + t_{j} + e_{ij}$$

where $y_{ij}$ are the BLUEs from the first stage, $\mu$ is the grand mean, and $g_{i}$ is the effect of the *i*th line that was modelled as random with ${\mathbf{g}} \sim\,N\left( {0, {\mathbf{I}}\sigma_{\text{G}}^{2} } \right)$ to estimate the genetic variance and as fixed to derive BLUEs for further genomic analyses. The effect of the *j*th trial $t_{j}$ was fixed, while the effect $e_{ij}$ that incorporated both the trial-by-line interaction variance and the residual effect was assumed random and followed a normal distribution with ${\mathbf{e}} \sim\,N\left( {0, {\mathbf{I}}\sigma_{\text{e}}^{2} } \right)$. All trials conducted across 2010–2012 were analysed together to create a single dataset of 415 lines used for training genomic prediction models, while the each year 2013–2016 was analysed separately to create four unique subpopulations of 164–185 lines that were used to validate these models. The subpopulations were unique in the sense that none of the lines occurred twice in different years and each line was assigned to the year of its first testing in multi-environment trials. All phenotypic analyses were conducted using the statistical package ASReml 3 (VSN International 2015) for the R programming environment (R development core team 2018).

### Genotypic data and population structure

DNA was extracted following the protocol by Saghai-Maroof et al. (1984) using leaf samples that were collected from F_4:5_ or doubled haploid lines by sampling a minimum of ten plants per line during early summer. All lines were genotyped using the DarT genotyping-by-sequencing (GBS) approach. Quality control was applied by filtering out markers with a call rate lower than 90%, a minor allele frequency smaller than 0.05, and more than 10% of missing data. Missing data of the remaining 7.3 K SNP markers were chromosome wise imputed by the *missForest* algorithm following Stekhoven and Bühlmann (2012), resulting on average in a coverage of one marker every 0.97 cM. The same marker data were again used for training genomic selection models with F_4:6_ lines. The minor change in average heterozygosity was expected to introduce a small error which was nevertheless seen to be acceptable considering the cost–benefit ratio of re-genotyping all lines in later generations. The population structure with the corresponding membership of each line to its subpopulation was investigated with a principal component analysis (Online Resource 1).

### Comparison of genomics-based, genomics-assisted, and phenotypic selection

The available genotypic and phenotypic data of the training dataset 2010–2012 and the four unique subpopulations 2013–2016 were combined and initially employed for investigating the merit of various prediction model extensions for genomic selection in early generations. The kinship between lines was for this purpose estimated by the genomic relationship matrix, which was computed according to the method described by Endelman and Jannink (2012):

2 $${\mathbf{K}} = {\mathbf{WW}}^{\text{T}} /2\varSigma \left( {p_{k} - 1} \right)p_{k}$$

where ${\mathbf{W}}$ is a centred N × M marker matrix of the *i* lines with $W_{ik} = Z_{ik} + 1 - 2p_{k}$ and $p_{k}$ being the allele frequency at the *k*th locus. The derived variance–covariance matrix was used to fit genomic best linear unbiased prediction models (GBLUP) and derive genomic estimated breeding values (GEBV):

3 $${\mathbf{y}} = {\mathbf{Xb}} + {\mathbf{Zg}} + {\mathbf{e}}$$

where ${\text{y}}$ is an N × 1 vector of BLUEs obtained in the phenotypic analysis and ${\mathbf{g}}$ is an N × 1 vector of additive effects with ${\mathbf{g}} \sim {\text{N}}\left( {0, {\mathbf{K}}\upsigma_{\text{G}}^{2} } \right)$ and the additive genetic variance $\sigma_{\text{G}}^{2}$ as well as its corresponding random effect design matrix ${\mathbf{Z}}$. The residual effect ${\mathbf{e}}$ followed a normal distribution with ${\mathbf{e}} \sim \,N\left( {0, {\mathbf{I}}\sigma_{\text{e}}^{2} } \right)$, and the fixed effect of the grand mean was contained in the vector ${\mathbf{b}}$ and its corresponding design matrix ${\mathbf{X}}$.

The genomic estimated breeding value for the *i*th line was defined by ${\text{GEBV}}_{i} = \mu + g_{i}$ with the additive genetic effect $g_{i}$ and the facultative constant $\mu$ designating the grand mean.

Given that genetic fingerprints are obtained at a breeding stage when preliminary yield trials are conducted in parallel (Guzmán et al. 2016; Gaynor et al. 2017), it could be worthwhile to integrate such early phenotypic information into genomic prediction models and in this way exploit the genetic relationship between the early and advanced generation lines to strengthen the predictiveness of preliminary yield trials (Endelman et al. 2014; Müller et al. 2015; Michel et al. 2017). This would give rise to a genomics-assisted selection in contrast to the genomics-based selection without any prior phenotypic information from the selection candidates. Model (3) was accordingly modified for this purpose with preliminary yield trials having a distinct fixed year effect from the years of multi-environment trials. However, it can be expected that the data quality from preliminary yield or observation trials will oftentimes be considerably low, thus introducing an appropriate weighting might be beneficial for achieving an optimal prediction ability. Heterogeneous residual variances were thus integrated into (3) by assigning a common residual variances to multi-environment trials that was though different from the one given to preliminary yield trials $\sum_{\text{e}} = {\text{diag}}\left( {\sigma_{{{\text{e}}_{\text{MET}} }}^{2} ,\sigma_{{{\text{e}}_{\text{PYT}} }}^{2} } \right)$ where $\sigma_{{{\text{e}}_{\text{MET}} }}^{2}$ and $\sigma_{{{\text{e}}_{\text{PYT}} }}^{2}$ are the residual variances for multi-environment trials and the respective preliminary yield trial.

**Fig. 1:**
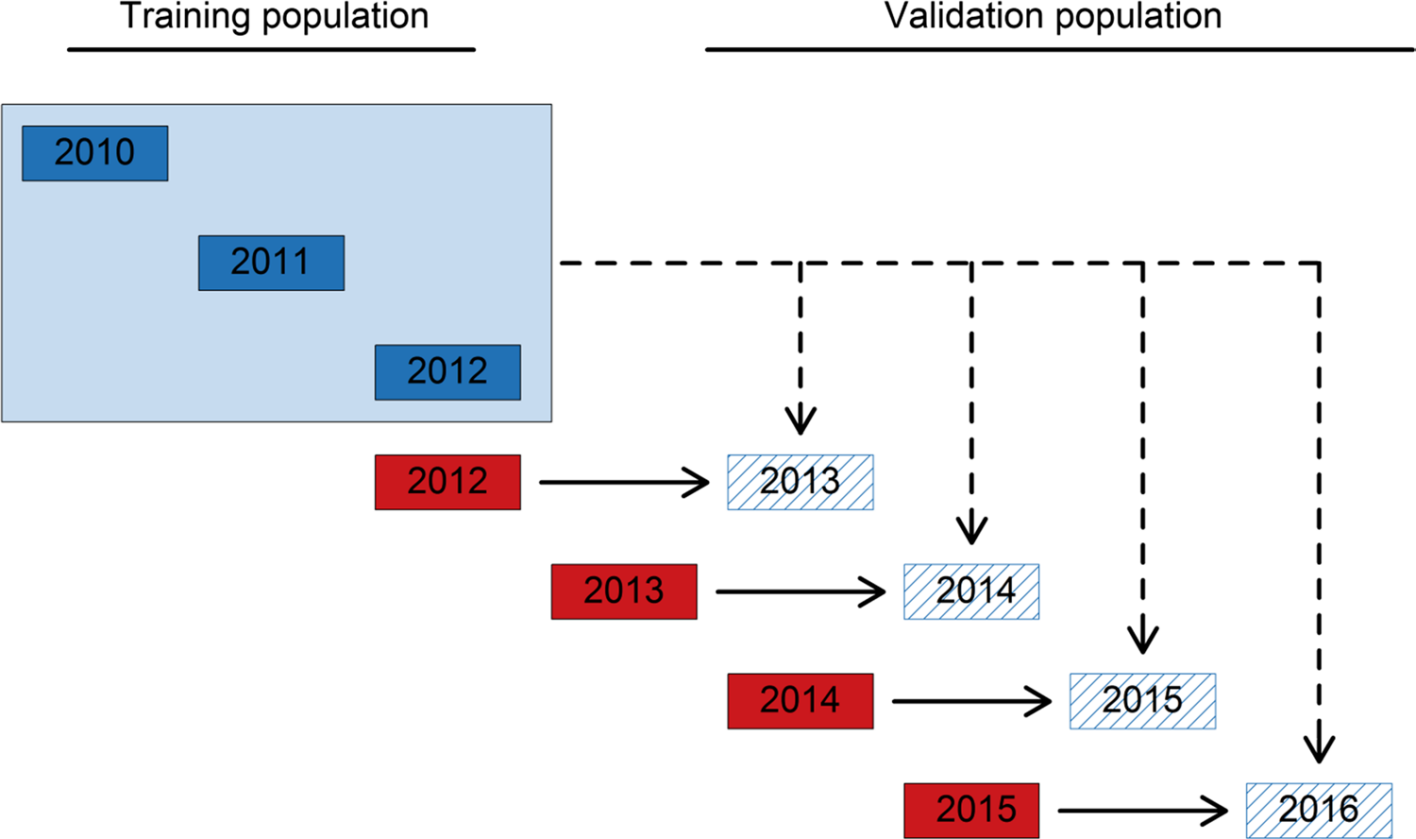
Forward prediction for the subpopulations tested in 2013–2016 (shaded) using lines tested in multi-environment trials 2010–2012 (blue) for genomics-based selection and lines from preliminary yield trials (red) for phenotypic selection (color figure online)

All models were examined in a forward prediction of lines tested in multi-environment trials 2013–2016 using 300 randomly sampled lines from the years 2010–2012 as training population for genomic-based selection and preliminary yield trials 2012–2015 for phenotypic as well as genomic-assisted selection (Fig. 1). A set of 100 unique lines was randomly sampled 30 times from each year of multi-environment trials 2013–2016, while the set sampled from preliminary yield trials was identical with the validation population, which finally resulted in 120 training by validation population combinations. All models for genomics-based and genomics-assisted selection were fitted with the mixed model package *sommer* (Covarrubias-Pazaran 2016) for R (R development core team 2018).

### Concepts for a simultaneous selection of grain yield and protein content

**Fig. 2:**
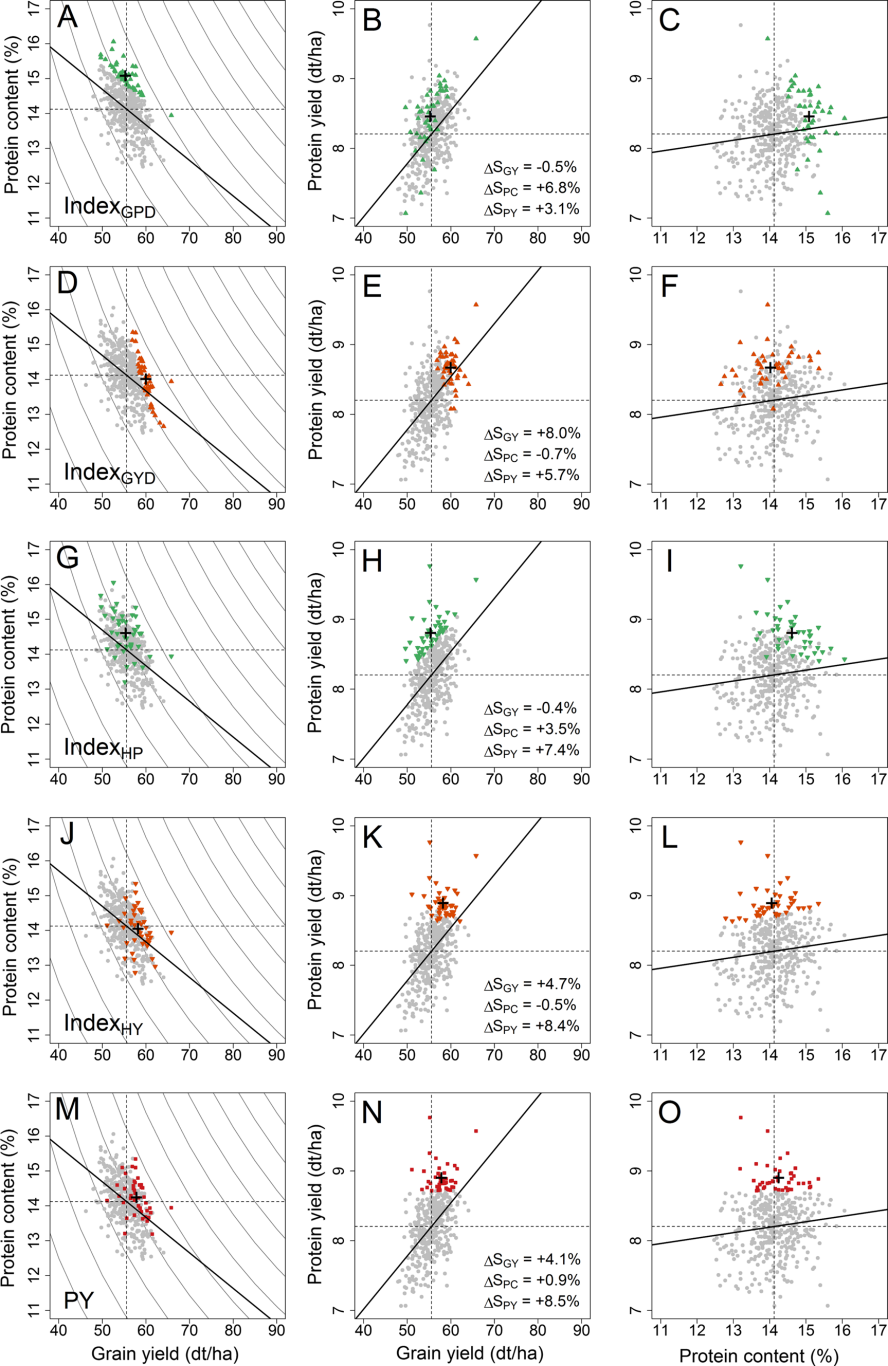
Illustration of the concepts for a simultaneous selection of grain yield and protein content on the 415 lines from 2010 to 2012 that were used as a training population for genomic prediction. The overall population averages for grain yield, protein content, and protein yield are indicated by the dashed lines, while isolines of equal protein yield are represented by solid grey lines. The concepts include selections based on grain protein deviations (**a**–**c**), grain yield deviations (**d**–**f**), high protein index (**g**–**i**), high yield index (**j**–**l**), and the protein yield (**m**–**o**). The 10% best performing lines according to each method are highlighted in colour, and their population average is displayed by a cross, which corresponds to the respective selection differential Δ*S* for grain yield (GY), protein content (PC), and protein yield (PY). Regression lines display the negative correlation between grain yield and protein content (*r* = − 0.47) and the positive correlation between protein yield and grain yield (0.53) as well as protein content (*r* = 0.11) (color figure online)

A strong negative correlation between grain yield and protein content could be observed with the available data in the study at hand (Fig. 2). Genotypes that deviate from this negative trend and constitute the highly appreciated outliers in breeding programmes were firstly identified by utilizing grain protein deviations that aim to point out genotypes with comparably high protein content at a given yield level (Monaghan et al. 2001). The simplest method for deriving these grain protein deviations by calculating the residuals from the regression of protein content on grain yield based on environmental means (Oury and Godin 2007) was chosen in this study. Hence, BLUEs were used in this study to obtain the residuals from a regression of protein content on grain yield (Fig. 2a–c):

4 $${\mathbf{GPD}} = {\mathbf{pc}} - \widehat{{{\mathbf{pc}}}} = {\mathbf{pc}} - \left( {\alpha + \beta {\text{g}}{\mathbf{y}}} \right) = {\mathbf{pc}} - \alpha - \beta {\mathbf{gy}}$$

with ${\mathbf{pc}}$ being the observed values for protein content, $\widehat{{{\mathbf{pc}}}}$ the protein content values predicted by the regression on grain yield, ${\mathbf{gy}}$ the observed grain yield, and finally with $\alpha$ and $\beta$ being the estimates for the intercept and regression coefficient, respectively. Changing the role of protein content and grain yield in the equation above brings forth grain yield deviations (Rapp et al. 2018) that were also considered as a viable selection criterion:

5 $${\mathbf{GYD}} = {\mathbf{gy}} - \widehat{{{\mathbf{gy}}}} = {\mathbf{gy}} - \left( {\alpha + \beta {\mathbf{pc}}} \right) = {\mathbf{gy}} - \alpha - \beta {\mathbf{pc}}$$

with $\widehat{{{\mathbf{gy}}}}$ being the predicted grain yield values from a regression of grain yield on protein content (Fig. 2d–f), and all other designations remaining constant. A closely related concept is the usage of a selection index of the form:

6 $${\mathbf{b}} = {\mathbf{P}}^{ - 1} {\mathbf{a}}$$

where ${\mathbf{b}}$ are the index weights, ${\mathbf{a}}$ is a vector of the index weights given by ${\mathbf{a}} = \left( {a_{\text{PC}} ,a_{\text{GY}} } \right)^{\text{T}}$, and ${\mathbf{P}}^{ - 1}$ is the inverse of the phenotypic variance–covariance matrix:

7 $$\left( {\begin{array}{*{20}c} {\sigma_{{{\text{P}}_{\text{PC}} }}^{2} } & {\sigma_{\text{P}} } \\ {\sigma_{\text{P}} } & {\sigma_{{{\text{P}}_{\text{GY}} }}^{2} } \\ \end{array} } \right)$$

where $\sigma_{{{\text{P}}_{\text{PC}} }}^{2}$ and $\sigma_{{{\text{P}}_{\text{GY}} }}^{2}$ are the phenotypic variance of the protein content and grain yield, respectively, and values $\sigma_{\text{P}}$ on the off-diagonal genetic represent the covariance between both traits. Aiming to improve protein content and holding grain yield stable, the index weights were chosen as $a_{\text{PC}} = 1$ and $a_{\text{GY}} = 0$ resulting in a vector of index weights given by:

8 $${\mathbf{b}} = \left( {\begin{array}{*{20}c} 1 \\ { - \sigma_{\text{P}} /\sigma_{{{\text{P}}_{\text{GY}} }}^{2} } \\ \end{array} } \right) = \left( {\begin{array}{*{20}c} { 1} \\ { - \beta } \\ \end{array} } \right)$$

and a restriction index of the form:

9 $${\mathbf{Index}}_{{{\mathbf{GPD}}}} = {\mathbf{pc}} - \beta {\mathbf{gy}}$$

where ${\mathbf{pc}}$ and ${\mathbf{gy}}$ were again the observed values for protein content and grain yield, thus highlighting the close connection between grain protein deviations as calculated in this study and a restriction index, which are in fact identical aside from the regression intercept ${\text{a}}$ that has though no influence in the ranking of line performances. Notice that the selection index (6) with the described index weights ${\mathbf{a}}$ is strongly related to a desired gain index in the form of a restriction index (Pesek and Baker 1969, 1970); however, the latter features a genetic correlation matrix, thus driving the genetic response to selection, while (6) and consequently the classical grain protein deviations would drive the phenotypic response to selection. Grain protein deviations represent thus a criterion for selecting high-protein lines with acceptable yield potential, while grain yield deviations and the according index

10 $${\mathbf{Index}}_{{{\mathbf{GYD}}}} = {\mathbf{gy}} - \beta {\mathbf{pc}}$$

seemed more suitable for developing high-yielding varieties, while maintaining the protein content (Fig. 2). A rather different concept that has been suggested for achieving a simultaneous gain for both protein content and grain yield is a selection based on protein yield (Simmonds 1995), which measures the total harvested seed nitrogen content. Regarding the isolines of equal protein yield (Fig. 2), it is clear that some lines possess a high protein yield due to high grain yield, whereas others realize it with elevated protein content, which results in a positive correlation with both protein content and, generally a stronger one, grain yield (Fig. 2m–o). Like the grain protein deviations, the protein yield is furthermore associated with nitrogen-use efficiency-related traits (Cormier et al. 2013) and was thus seen as a further important target criterion in this study.

The possibility to combine both concepts was subsequently explored by utilizing the deviations from a linear regression of protein yield on either grain yield (Fig. 2g–i) or protein content (Fig. 2j–l). Lines that show a high performance, i.e. positive deviation, in the former exhibit a high protein yield due to high protein content, and positive deviations in the latter indicate a superior protein yield caused by grain yield. Both cases were again expressed in the form of restriction indices:

11 $${\mathbf{Index}}_{{{\mathbf{HY}}}} = {\mathbf{py}} - \beta {\mathbf{pc}}$$

and

12 $${\mathbf{Index}}_{{{\mathbf{HP}}}} = {\mathbf{py}} - \beta {\mathbf{gy}}$$

with ${\mathbf{py}}$ protein yield, ${\mathbf{gy}}$ for grain yield, ${\mathbf{pc}}$ for protein content, and $\beta$ being again the index weight for either grain yield or protein content and can be again interpreted as the regression coefficient. The first index ${\mathbf{Index}}_{{{\mathbf{HY}}}}$ will be designated as the high yield index, while the second index ${\mathbf{Index}}_{{{\mathbf{HP}}}}$ is referred to as the high protein index in this study in order to differentiate them from the other two indices ${\mathbf{Index}}_{{{\mathbf{GPD}}}}$ and ${\mathbf{Index}}_{{{\mathbf{GYD}}}}$. All indices were calculated based on phenotypic data and used for phenotypic selection with preliminary yield trial. Notwithstanding, the above-described concepts are representing steps towards a simultaneous selection of grain yield and protein content; it was argued that in the case of genomic selection, the genomic correlation (de los Campos et al. 2015; Gianola et al. 2015) between traits has to be taken into account especially as the genomic and genetic correlation converge if markers adequately capture all genetic information. Hence, using a genomic variance–covariance matrix instead of a phenotypic variance–covariance matrix might be more suitable to drive the response to selection in an index selection, and several genomic selection indices were developed by using the same methodological approaches as described above:

13 $${\mathbf{b}} = {\mathbf{G}}^{ - 1} {\mathbf{a}}$$

where ${\mathbf{b}}$ are the genomic index weights, ${\mathbf{G}}^{ - 1}$ is in this case the inverse of the genomic variance–covariance matrix, and ${\mathbf{a}}$ is again the vector of the desired gains with ${\mathbf{a}} = \left( {1, 0} \right)^{\text{T}}$ in order to improve the primary trait and holding the secondary trait stable. The matrix ${\mathbf{G}}$ was for simplicity derived from the Pearson correlation between the GEBVs of the involved traits in order to test the computational least demanding option suited to large datasets generated in applied breeding programmes:

14 $${\mathbf{G}}_{{{\mathbf{cor}}}} = \left( {\begin{array}{*{20}c} {\sigma_{{{\text{GEBV}}_{i} }}^{2} } & {\sigma_{\text{GEBV}} } \\ {\sigma_{\text{GEBV}} } & {\sigma_{{{\text{GEBV}}_{j} }}^{2} } \\ \end{array} } \right)$$

where $\sigma_{{{\text{GEBV}}_{i} }}^{2}$ and $\sigma_{{{\text{GEBV}}_{j} }}^{2}$ are the variance of the GEBVs for traits involved in the respective index, and $\sigma_{\text{GEBV}}$ on the off-diagonal genetic represents the covariance between both traits. The according genomic selection indices can be as well interpreted in terms of deviations from the regression line; though in contrast to the phenotypic indices, they are representing residuals from a regression of the GEBVs from grain yield, protein content, and protein yield against each other. The presented methods for a simultaneous selection of grain yield and protein content were finally tested with the models for phenotypic, genomics-based, and genomics-assisted selection in a forward prediction across years as described in the previous section (Fig. 1).

### Breeding strategies and selection gain

The prediction accuracy of phenotypic selection and all genomic models was assessed by the correlation between genomic estimated breeding values as well phenotypic records from preliminary yield trials with the observed phenotypic values for the agronomic traits and the selection indices in the validation populations divided by the square root of the heritability. For this purpose, a genomic heritability was estimated for each trait and resampling of the validation populations in 2013–2016 by

15 $${\text{h}}_{\text{GEN}}^{2} = (\sigma_{\text{P}}^{2} - \sigma_{\text{e}}^{2} )/\sigma_{\text{p}}^{2}$$

where $\sigma_{\text{P}}^{2}$ is the phenotypic variance of investigated trait and $\sigma_{\text{e}}^{2}$ the error variance obtained from a GBLUP model that only contained phenotypic data from the validation population. Aside from assessing the prediction accuracy, it was of further interest to investigate how these estimates would translate into a response to selection across years. The 10–50% best performing lines among the 100 selection candidates of each resampling step were therefore selected according to grain yield, protein content, protein yield as well as the above-described phenotypic and genomic selection indices. For this purpose, the predicted average performance of the selected fraction of lines for each individual trait was estimated by:

16 $$\hat{\mu }_{{{\text{Sel}}_{i} }} = \mu_{i} + {\text{h}}_{{{\text{GEN}}_{i} }}^{2} \left( {\mu_{{{\text{Sel}}_{i} }} - \mu_{i} } \right)$$

where $\mu_{i}$ is the average trait performance of an entire validation population, $\mu_{{{\text{Sel}}_{i} }}$ is the average trait performance of the selected lines, and ${\text{h}}_{{{\text{GEN}}_{i} }}^{2}$ is the genomic heritability of the *i*th trait. Selection decisions were subsequently verified by the average line performance across the multi-environment trials in the validation year by:

17 $$R_{{{\text{Rel}}_{i} }} = \left( {\hat{\mu }_{{{\text{Sel}}_{i} }} - \mu_{i} } \right)/\mu_{i}$$

where ${\text{R}}_{{{\text{Rel}}_{i} }}$ is the predicted relative response to selection for the *i*th trait, $\mu_{i}$ is the population mean of the validation population in the validation year, and $\hat{\mu }_{{{\text{Sel}}_{i} }}$ is the predicted mean of the selected population of best performing lines. Although selection decisions were based on various methods, the main interest was the assessment of the direct or indirect response to selection for grain yield, protein content, and protein yield. Finally, a combined breeding strategy was tested by splitting selection decisions of the 10% best performing lines into two halves, with the first half of lines (5%) being selected with a protein content index and the other half with a grain yield index resulting in a broad range for all involved traits among the selected candidates. Combining both strategies corresponded thus to an evaluation of genomic breeding when developing both high-protein genotypes with acceptable yield potential as well as high-yielding varieties with sufficient protein content that is common in wheat breeding for offering a portfolio of varieties from multiple quality classes to farmers.

## Results

### Models for genomics-assisted line breeding

The prediction accuracy for grain yield using the phenotypic data from preliminary yield trials was low (*r* = 0.25) and was vastly surpassed by the one for genomics-based selection (*r* = 0.45) (Table 1). The inverted situation was though observed for the highly heritable protein content where phenotypic selection had a prediction accuracy of *r* = 0.60, while a genomics-based selection strategy could merely achieve *r* = 0.53. A large improvement could be achieved by including prior information from preliminary yield trials into the prediction models, which resulted in a genomics-assisted selection with an average prediction accuracy of *r* = 0.47 for grain yield that surpassed the prediction accuracy for phenotypic selection by 88%. Similar results were obtained for the protein content, where the employment of genome-wide marker data to improve the phenotypic data from preliminary yield trials gave an accuracy of *r* = 0.69, which surpassed the best previous method, i.e. phenotypic selection by 15%.

**Table 1:** Comparison between different selection methods for a simultaneous selection of yield and quality in terms of prediction accuracy across years for grain yield (GY), protein content (PC), protein yield (PY), and the restriction indices for a simultaneous selection of protein content and grain yield

Predictor trait	Method	Prediction accuracy						
		GY	PC	PY	Index_GPD_	Index_GYD_	Index_HP_	Index_HY_
Grain yield	Phenotypic	0.25	− 019	0.10	− 0.10	0.23	− 0.12	0.15
	Genomics-based	0.45	− 0.37	0.12	− 0.23	0.34	− 0.24	0.28
	Genomics-assisted	0.47	− 0.41	0.10	− 0.28	0.35	− 0.29	0.23
Protein content	Phenotypic	− 0.38	0.60	0.20	0.56	− 0.10	0.54	− 0.03
	Genomics-based	− 0.38	0.53	0.12	0.48	− 0.14	0.45	− 0.10
	Genomics-assisted	− 0.50	0.69	0.16	0.62	− 0.18	0.59	− 0.11
Protein yield	Phenotypic	0.00	0.20	0.20	0.26	0.17	0.21	0.13
	Genomics-based	0.18	0.13	0.33	0.25	0.32	0.22	0.30
	Genomics-assisted	0.13	0.19	0.36	0.30	0.31	0.26	0.29
Index_GPD_^a^	Phenotypic	− 0.37	0.58	0.19	0.55	− 0.07	0.52	− 0.03
	Genomics-based	− 0.17	0.41	0.22	0.43	0.05	0.39	0.07
	Genomics-assisted	− 0.34	0.60	0.24	0.58	− 0.03	0.55	0.01
Index_GYD_^b^	Phenotypic	0.22	− 0.14	0.10	− 0.06	0.22	− 0.09	0.15
	Genomics-based	0.30	− 0.08	0.23	0.05	0.33	0.01	0.28
	Genomics-assisted	0.21	0.00	0.24	0.11	0.30	0.08	0.25
Index_HP_^c^	Phenotypic	− 0.30	0.53	0.22	0.52	− 0.02	0.48	0.01
	Genomics-based	0.00	0.31	0.33	0.39	0.21	0.36	0.22
	Genomics-assisted	− 0.08	0.41	0.35	0.48	0.19	0.44	0.20
Index_HY_^d^	Phenotypic	0.25	− 0.16	0.11	− 0.07	0.25	− 0.11	0.17
	Genomics-based	0.26	0.02	0.32	0.16	0.35	0.13	0.32
	Genomics-assisted	0.26	0.02	0.32	0.15	0.36	0.12	0.32

Prediction of the indices was based on a genomic variance–covariance matrix for genomics-based and genomics-assisted selection, while phenotypic selection and validation was based on a phenotypic variance covariance matrix

^a^Restriction index for holding grain yield stable and increasing the protein content

^b^Restriction index for holding protein content stable and increasing the grain yield

^c^Restriction index for holding grain yield stable and increasing the protein yield

^d^Restriction index for holding protein content and increasing the protein yield

### Multi-trait selection for grain yield and protein content

The phenotypic and genomic prediction models were subsequently assessed for their potential in achieving the goal of conducting a simultaneous selection for grain yield and protein content. For this purpose, the four restriction indices as well as protein yield were subsequently tested in a forward prediction for testing their merit in presence of strong genotype-by-environment interaction when predicting across years as well as the influence of genetic relationships when predicting across subpopulations derived from sets of different crosses. Phenotypic selection with protein yield data from preliminary yield trials and genomics-based selection methods had a positive prediction accuracy for both protein content and grain yield (Table 1). It was, moreover, evident that phenotypic selection with restriction indices and phenotypic variance–covariance matrix was far from optimal to target the protein content/grain yield trade-off as, e.g., the grain protein deviation still showed a negative correlation of *r* = − 0.37 with yield which was merely slightly higher than when directly using the protein content for prediction (*r*_PC;GY_ = − 0.38). Additionally, all investigated restriction indices had a lower prediction accuracy when aiming to improve the protein yield by phenotypic selection in preliminary yield trials in comparison with genomics-based selection (− 94%), which expended even more for a genomics-assisted selection approach (− 105%). Genomics-assisted selection based on protein yield per se was furthermore superior than using restriction indices both in an early generation phenotypic selection or genomic-assisted selection. The difference in accuracy with a genomics-assisted selection on protein yield per se and the genomic selection indices was relatively large both for the grain protein deviations (− 50%) and grain yield deviations (− 49%) indices. The high protein and yield indices showed on the other hand much more subtle difference with a prediction of protein yield per se that amounted to − 3% and − 15%, respectively. The high protein index was furthermore accompanied by a strong adjustment for grain yield (*r* = − 0.08) in comparison with predictions based on protein content per se (*r*_PC;GY_ = − 0.50), but still achieved an adequate prediction accuracy for protein content (*r* = 0.41) that was though again smaller than by pure genomics-assisted selection on protein content (*r* = 0.69). Forward prediction with the complementary high yield index resulted analogously in a comparably low accuracy for yield (*r* = 0.26) and protein yield (*r* = 0.32) due to the generally lower prediction accuracy for grain yield (*r* = 0.47) as well as the large negative trade-off with the protein content (*r*_GY;PC_ = − 0.41).

**Fig. 3:**
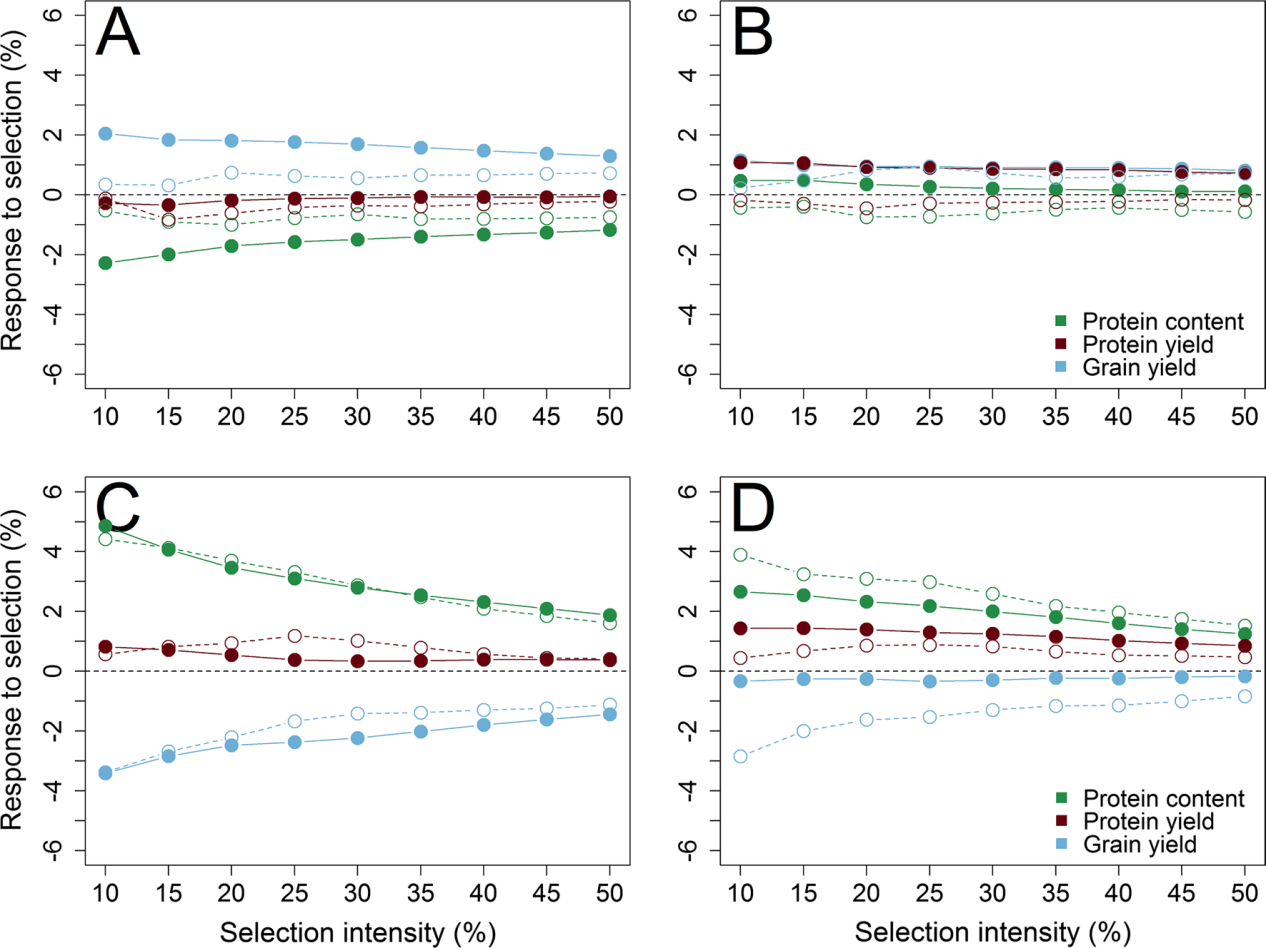
Response to selection for grain yield, protein content, and protein yield based on grain yield per se (**a**) and the high yield index (**b**); protein content (**c**) per se and the high protein index (**d**) as dependant variables in genomic-assisted (closed circles) forward predictions and for phenotypic selection with preliminary yield trial data (open circles)

This large trade-off was also reflected by the response to genomic-assisted selection for grain yield at a selection intensity of 10% which amounted to 2.0%, which was albeit associated with a severe loss in quality by reducing protein content relatively by − 2.3% (Fig. 3a). Selecting with the high yield index was able to prevent this loss by keeping the protein content stable (Δ_PC_ = 0.5%) and at the same time giving a large response for grain yield (Δ_GY_ = 1.2%) and protein yield (Δ_PY_ = 1.1%) (Fig. 3b). Response to genomics-assisted selection with the grain yield deviations gave similar results for grain yield (Δ_GY_ = 1.0%); the selection response for protein content (Δ_PC_ = − 0.1%) and protein yield (Δ_PY_ = 0.5%) was though markedly smaller (Online Resource 2). The response for genomics-assisted selection was furthermore much higher than the one from phenotypic selection when aiming at the selection of high-yielding lines and holding the protein content stable, and this clear difference could likewise be observed for a breeding strategy that aimed to increase the protein content and preserve an acceptable yield potential (Fig. 3d). The higher prediction accuracy of genomics-assisted selection (*r* = 0.69) in comparison with phenotypic selection (*r* = 0.60) for the protein content also inflated the trade-off with grain yield and hampered the effectiveness of the according restriction indices (Table 1); despite the adjustment, a negative response to grain yield was still present using grain protein deviation (Δ_GY_ = − 1.4%) as well as the high protein index (Δ_GY_ = − 0.3%) at a selection intensity of 10%. However, the response to protein yield was still higher with genomic-assisted selection (Δ_PY_ = 1.1%) than by phenotypic selection (Δ_PY_ = 0.4%).

### Complementing breeding strategies with genomic selection indices

**Fig. 4:**
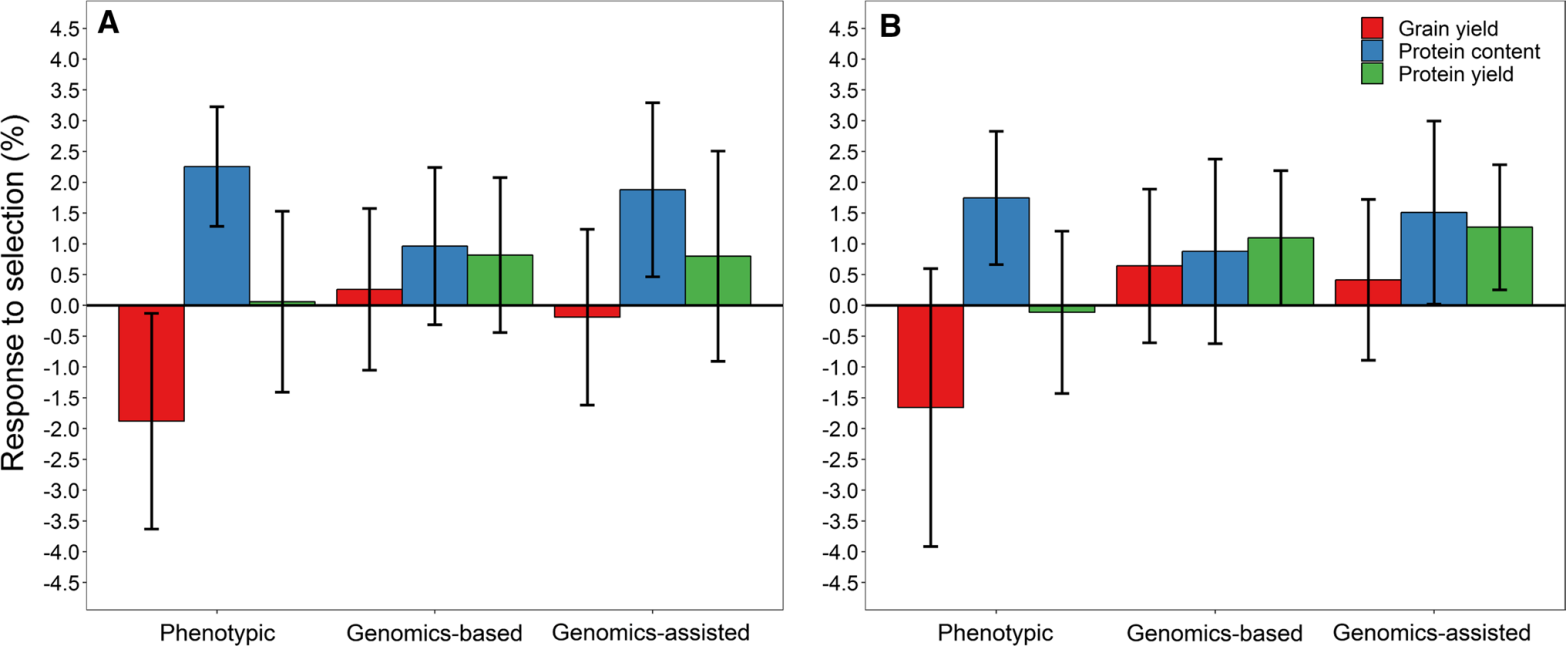
Mean and standard deviations of the response to selection for grain yield (GY), protein content (PC), and protein yield (PY) for phenotypic, genomic-based and genomics-assisted selection with grain yield and protein deviations (**a**) as well as high yield and protein indices (**b**) obtained from replicated forward predictions of 2013–2016. The response to selection when selecting the 10% best performing lines by either method is displayed, where half of lines (5%) being selected with a protein content index and the other half with a grain yield index

The restriction indices that achieved a genetic improvement for the protein content or grain yield and at the same time hold the other trait stable were largely independent from each other, which could be confirmed by the low prediction abilities when, e.g., predicting grain yield deviations by grain protein deviations (Table 1). Hence, both breeding strategies are complementary and in order to develop a portfolio of varieties from multiple quality classes with acceptable yield potential, the parallel usage of the respective selection indices and the overall response to selection by such a strategy were finally investigated. For this purpose, half of the 10% best performing lines were selected with an index aiming at a superior yield potential, while maintaining the protein content and the other half with the analogous index for identifying lines that combine high protein content with an acceptable grain yield. Using data from preliminary yield trials for coming to such a combined selection decision with grain protein and yield deviations gave some positive response to protein content (Δ_PC_ = 2.3%) and protein yield (Δ_PY_ = 0.1%) led though on average to reduction in grain yield (Δ_GY_ = − 1.9%) (Fig. 4a).

Notice that the usage of grain protein and yield deviations as well as the high protein and yield indices in a phenotypic selection based on data from preliminary yield trials achieved merely a marginal reduction in the protein content/grain yield trade-off, which was also evident by regarding the prediction ability for these indices in a phenotypic selection (Table 1). On the other hand, large differences between the different indices and concepts were observed in genomics-based and genomic-assisted selection, in which a combination of high yield and protein indices gave a +47% higher relative response to selection for protein yield that was accompanied by a threefold higher response to grain yield in comparison with grain protein and yield deviations (Fig. 4b).

## Discussion

This study concentrated on different breeding methods to achieve a simultaneous response to selection for grain yield and protein content in conventional and genomics wheat breeding. The merit of various prediction models for an early generation genomic and phenotypic selection was firstly compared among each other. These methods were subsequently used to assess the potential and limits of different concepts for addressing the well-known negative trade-off between grain yield and protein content, which substantially influences the overall response to selection as revealed in the forward prediction across multiple years in the investigated applied wheat breeding programme.

### Model extensions for genomic-assisted line breeding

The implementation of genomic selection into the breeding scheme of a conventional line breeding programme showed high potential and performed sustainably better for grain yield than phenotypic selection based on one single plot of a given year as conducted in preliminary yield trials. Classical phenotypic selection even with low-quality data can nevertheless outperform genomic selection as seen for highly heritable protein content, whereas selection on seemingly high-quality phenotypic data obtained from multiple trials might on the other hand be less suitable than genomic selection to reliably identify lines with high potential across many environments and years (Belamkar et al. 2018). One reason for this observation could be that the presence of genotype-by-environment interaction causes a continuous shift back and forwards with regard to the actual selection goal when using pure phenotypic selection as it most times takes merely the observed performance in the current year into account (Gaynor et al. 2017). Combining the advantage of phenotypic selection based on preliminary yield trials with prior information about line performance and genomics-based selection that utilizes data from multiple years in a genomics-assisted selection resulted in higher prediction abilities and also increased their stability with 31% and 20% smaller standard deviations for protein content and grain yield, respectively. Genomics-assisted selection performed furthermore in more than 75% of cases better than either pure phenotypic or genomics-based selection, and recycling the once obtained marker data by modelling genomic relationship in multi-environment trials has the potential to improve phenotypic data especially for low heritable traits or costly to phenotype traits that are only assessed in few trials such as quality-related traits (Fiedler et al. 2017; Hayes et al. 2017; Kristensen et al. 2018). According simulations suggested that the relative advantage of integrating genomic selection into a conventional breeding scheme in the form of a two-stage genomic-assisted selection in F_5_ preliminary yield trials followed by F_6_ multi-environment trials was 54% for grain yield, 7% for protein content, and 32% for protein yield in response to selection when compared with two-stage phenotypic selection using data from the particular wheat breeding programme of this study (unpublished data). Notwithstanding, there is even more upward potential in genomic breeding by routinely planning crosses with marker data combined with an earlier crossing of the most promising parents to shorten generation cycles (Lehermeier et al. 2017; Osthushenrich et al. 2017; Müller et al. 2018) as well as fast recurrent genomic selection schemes in a two-part strategy with a rapid population improvement cycle and a separate variety development part (Gaynor et al. 2017; Gorjanc et al. 2018).

### Genomic predictions for finding and creating outliers

One major aspect when integrating genomic selection either in conventional breeding scheme or following a two-part breeding strategy is though the simultaneous selection for multiple traits, which is readily feasible if traits show favourable genetic correlations such as dough quality and protein content in bread and spelt wheat (Battenfield et al. 2016; Rapp et al. 2017) but a lot more challenging with traits displaying unfavourable correlations such as the well-known trade-off between grain yield and protein content (McNeal 1982; Simmonds 1995) that was more detailed investigated in the second half of this study. Potential causes for this relationship are a dilution effect of nitrogen allocated to an increasing number of kernels conferring higher grain yields (Acreche and Slafer 2009) as well as the competition between carbon and nitrogen for energy (Munier-Jolain and Salon 2005). Although a strong negative genetic correlation is frequently observed between grain yield and protein content in wheat (Laidig et al. 2017; Thorwarth et al. 2018), environmental influences can extensively alter the magnitude of this negative relationship (Oury and Godin 2007) making it necessary to test genotypes in multi-environment trial networks to enable a simultaneous selection of both traits (Oury et al. 2003).

Testing in multi-environment trial networks is though only feasible in advanced generations with relatively few pre-selected genotypes making a marker-assisted selection in early generations an attractive alternative approach. The implementation of a successful marker-assisted selection requires first of all elucidating the underlying trait genetic architecture, and numerous marker loci have been identified causing the trade-off between grain yield and protein content either due to pleiotropy or close linkage of causal QTL rendering an independent improvement difficult (Blanco et al. 2012; Wang and Cui 2012; Bogard et al. 2013; Zhao et al. 2017). Nevertheless, some QTL have been described that increase the protein content independent or with a marginal impact on grain yield (Blanco et al. 2012; Wang and Cui 2012), amongst them the prominent *Gpc*-*B1* gene from wild emmer (Uauy 2006; Tabbita et al. 2017) that has also been introgressed into elite durum and bread wheat backgrounds (Brevis and Dubcovsky 2010; Eagles et al. 2014). Although some genetic improvement is feasible by using these identified QTL in a marker-assisted selection its potential will be limited due to the focus on rather low number of loci leading to the fast fixation of favourable alleles. Hence, genomic selection that targets many loci both with minor and major effect might be more suitable to achieve a higher response to selection in the long-term. Using a large number of genome-wide distributed markers for such an approach would nevertheless encompass both loci that show favourable effect, i.e. improvement in either protein content or grain yield without a negative effect on other traits but also unfavourable loci that potentially cause the protein content/grain yield trade-off.

Addressing this issue, a desired gain index (Pesek and Baker 1969) for genomic selection was employed restricting either the protein content or grain yield and in this way preferably increase the allele frequency of favourable loci that confer an increase in grain yield or protein content without negatively influencing the respective other trait. Aside from largely targeting these loci, holding the population average for one of the traits stable also eased the identification of lines with favourable allele combinations that possess an elevated grain yield with average protein content and increased protein yield even in the presence of strong negative genomic correlation between protein content and grain yield. Preliminary investigations using cross-validation with these genomic selection indices did not show any benefit of multivariate models to derive variance–covariance matrices that contain this genomic correlation for calculating appropriate index weights. Furthermore, no added value was observed of using a closely related method that derives genomic selection indices by multiplying the vector of genomic estimated breeding values with the genomic relationship matrix (Ceron-Rojas et al. 2015), while a Smith–Hazel index aiming to maximize the net merit (Smith 1936; Hazel 1943) did not lead to desired gain, i.e. maintaining grain yield or protein content but favoured one trait at cost of the other. Accordingly, it can be recommended to focus on genomic selection indices that correspond to deviations from regression line when conducting a simultaneous selection for grain yield and protein content and for finding the desirable outliers from the common trend, although it should be noticed that other methods such as using the multi-optimization framework by setting optimal compromise solutions or from the Bayesian decision theory have also shown great promise (Akdemir et al. 2018; de Villar-Hernández et al. 2018).

Identification of these outliers is of high interest to breeders, and especially, the grain protein deviation has received large attention (Monaghan et al. 2001) and has even become a major criterion for variety registration in France (F. Löschenberger pers.comm.). It is, moreover, associated with post-anthesis nitrogen uptake in bread (Bogard et al. 2010; Latshaw et al. 2016) and durum wheat (Suprayogi et al. 2011) (Table 2). This suggested that selecting genotypes that show superior performance in the genomic selection index based on grain protein deviations potentially enables an indirect selection for a difficult to phenotype trait, which might lead to an indirect genetic improvement for this important component of nitrogen-use efficiency in a genomic breeding approach. The underlying genetic base of these deviations from the regression line is furthermore highlighted by a larger grain protein deviation of hybrid wheat in comparison with line varieties (Thorwarth et al. 2018) that might also be influenced by a different root architecture to improve nitrogen uptake (Cormier et al. 2016; Hawkesford 2017) and supposedly causes a larger yield stability for some genotypes (Mühleisen et al. 2014; Liu et al. 2017). Accordingly, several QTL related to grain protein deviation have been mapped in wheat amongst others in the proximity of major genes like *Ppd*-*D1* regulating photoperiodic sensitivity and the semi-dwarfing genes *Rht*-*B1* and *Rht*-*D1* (Cormier et al. 2014; Guttieri et al. 2017) with some candidate genes being identified (Habash et al. 2007; Li et al. 2011). Notwithstanding, polygenic inheritance with a genetic architecture of many small to medium effect loci renders the reliable identification of genotypes with large positive grain protein deviation difficult in the framework of genotype-by-environment interaction making variety testing in multi-environment trials necessary (Oury and Godin 2007), which can additionally be supported by prediction models that characterize environments with respect to nitrogen stress (Ly et al. 2017). No large benefit was though seen for genomic breeding using grain protein deviations as a selection index in this study when employing the basic GBLUP model in a forward prediction across years, especially with respect to compensate the protein content/grain yield trade-off. Hence, the response to selection for total seed nitrogen yield given by protein yield was relatively seen substantially lower with grain protein deviations than by utilizing the high protein index that aimed to maximize protein yield by the protein content while holding grain yield stable. The latter index had additionally a marked advantage both in genomic and phenotypic selection in early generations, while it likewise had some relationship with post-anthesis nitrogen uptake (Table 2).

**Table 2:** Phenotypic correlation of protein yield and the presented selection indices with post-anthesis nitrogen uptake and remobilization as well as protein content and grain yield in wheat

	Protein yield	Index_GPD_^a^	Index_GYD_^b^	Index_HP_^c^	Index_HY_^d^
Nitrogen remobilization	0.37	0.19	0.43	0.17	0.43
Nitrogen uptake	0.34	0.43	0.22	0.45	0.23
Protein content	0.27	0.73	0.00	0.72	0.00
Grain yield	0.54	0.00	0.73	0.00	0.72

Performance estimates as reported by Bogard et al. (2010) and Latshaw et al. (2016) were used to derive the respective selection indices, while for durum, wheat values were averaged over the three environments reported by Suprayogi et al. (2011). The respective correlation coefficients obtained from the individual studies were subsequently averaged over all three studies

^a^Restriction index for holding grain yield stable and increasing the protein content

^b^Restriction index for holding protein content stable and increasing the grain yield

^c^Restriction index for holding grain yield stable and increasing the protein yield

^d^Restriction index for holding protein content and increasing the protein yield

A genetic improvement in post-anthesis nitrogen uptake seems thus to be possible by selecting lines outstanding in the grain protein deviation and high protein indices, whereas the obtained results indicated that selecting lines with superior performance in the grain yield deviation and high yield indices will lead to a more pronounced gain in nitrogen remobilization that could also be a desirable trait when breeding for adaption to regions with dry conditions in late season limiting post-anthesis nitrogen uptake (Hawkesford 2014). The compensation of the above-described dilution effect by lines with high protein yield caused by grain yield while maintain protein content suggested furthermore a different genetic control of grain yield deviations (Rapp et al. 2018), while a genomic breeding approach showed considerable advantage for achieving a high total seed nitrogen yield and nearly completely removed the negative trade-off in this study. The mentioned negative correlation between grain or seed yield and protein content in wheat has been observed in many small-grain cereal species and legumes (Simmonds 1995; Munier-Jolain and Salon 2005) and breeding for a high protein yield either via grain yield or protein content might also constitute useful concepts for the genetic improvement in other crops like soybean (Kurasch et al. 2017). Both represent complementary selection strategies exploiting the principle that protein yield is correlated both with nitrogen uptake and remobilization each causing a superior total seed nitrogen yield (Cormier et al. 2013). Although no totally clear differentiation between both traits is feasible with the here presented genomic selection indices, the results from this and other studies (Bogard et al. 2010; Suprayogi et al. 2011; Latshaw et al. 2016) indicate that the presented restriction indices might aim at a more directed improvement in specific traits related to nitrogen-use efficiency than selecting on protein yield per se. Selection indices constitute thus valuable tools to support selection decisions either in genomic or conventional breeding schemes in order to ease the identification of genotypes with desirable trait combination among several thousands of selection candidates with respect to yield, quality, tolerances, and resistances. This will facilitate the development of new varieties with potentially higher nutrient use efficiency that are, moreover, well adapted to changing growing conditions, which represents one of the major columns of agricultural plant production comprising plant breeding, physiology, protection, and agronomic practices (Bodin Dresbøll and Thorup-Kristensen 2014; Hellemans et al. 2018).

## Conclusions

This study investigated the potential and limits of a simultaneous selection for grain yield and protein content in genomic wheat breeding. Model extensions for genomic selection enabled an increase in the prediction accuracy for the individual traits as well as the employed genomic selection indices, particularly by combining phenotypic and genomic information for a genomics-assisted selection approach in preliminary yield trials. Genomic selection indices that corresponded to deviations from the common regression of the involved traits were most promising for handling the observed strong negative phenotypic and genomic correlations, especially when aiming to maximize protein yield either via an elevated grain yield or protein content while holding the population average for the other respective trait stable. Forward prediction across years employing these concepts revealed that a strong response to selection for protein yield, i.e. total seed nitrogen content could be achieved by genomic breeding combining both selection strategies, which suggested that it is feasible to develop varieties that combine superior yield potential with comparably high protein content, thus utilizing available nitrogen resources more efficiently.

### Author contribution statement

SM wrote the manuscript and analysed the data. CA supported in the statistical analysis. FL, BP, and ES designed the field trials and collected the phenotypic data in the field. FL and HB initiated and guided through the study. All authors read and approved the final manuscript.

## Electronic supplementary material

Below is the link to the electronic supplementary material.

Supplementary material 1 (PDF 179 kb)

Supplementary material 2 (PDF 163 kb)
