# Supplementary material for: Simultaneous selection for grain yield and protein content in genomics-assisted wheat breeding
Source: Theor Appl Genet. 2019 Feb 27;132(6):1745–60. doi: 10.1007/s00122-019-03312-5 (PMC6531418; doi:10.1007/s00122-019-03312-5)
Supplement: Supplementary file 2 — Supplementary material 2 (PDF 163 kb) [file 122_2019_3312_MOESM2_ESM.pdf]

Figure S2

Article Title: Simultaneous selection for grain yield and protein content in genomics-assisted wheat breeding

Journal: Theoretical and Applied Genetics

Authors: Sebastian Michel, Franziska Löschenberger, Christian Ametz, Bernadette Pachler, Ellen Sparry, Hermann Bürstmayr

Name, affiliation, and email of corresponding author:

Sebastian Michel
Department for Agrobiotechnology (IFA-Tulln)
Institute for Biotechnology in Plant Production
University of Natural Resources and Life Sciences, Vienna (BOKU)
Konrad-Lorenz-Str. 20, 3430 Tulln, Austria
e-mail: sebastian.michel@boku.ac.at

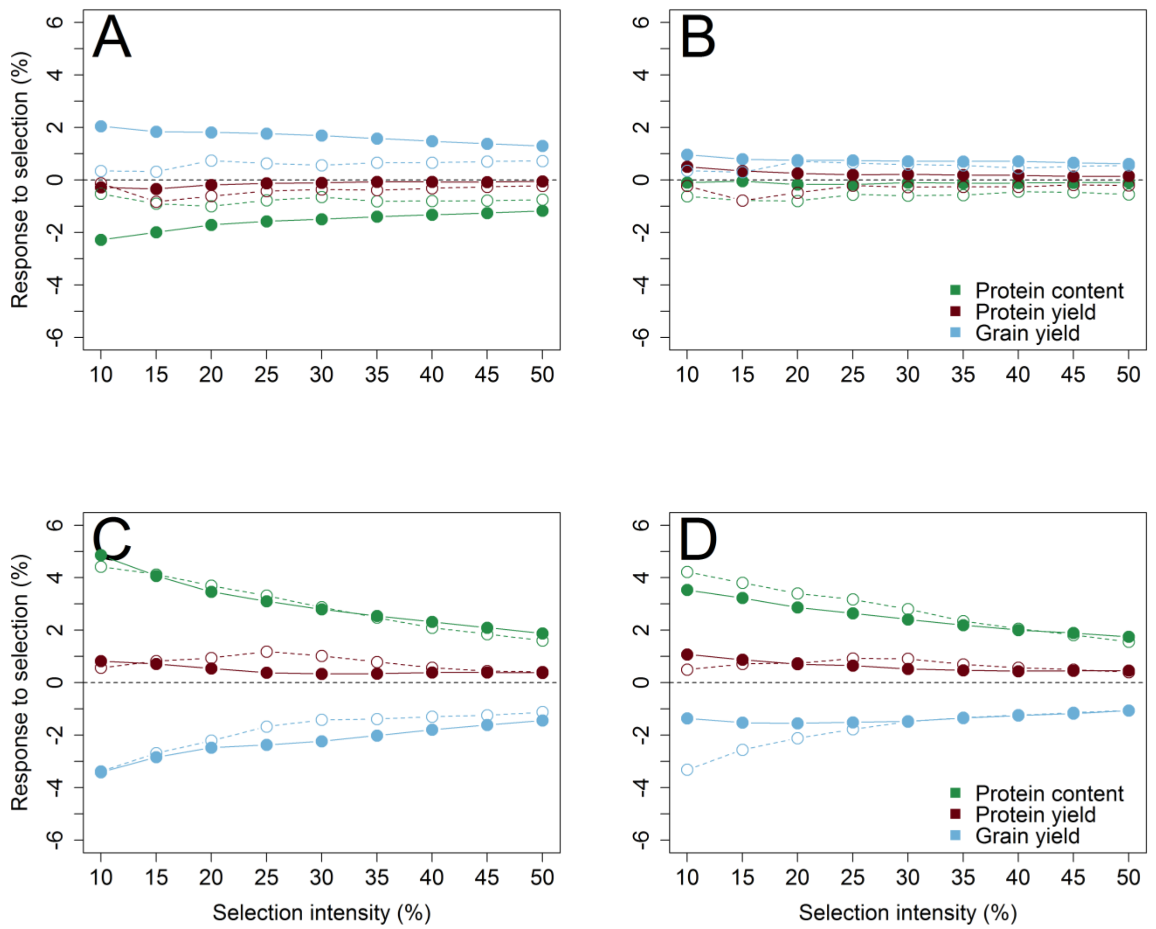

Online resource 2 Fig. S2 Response to selection for grain yield, protein content, and protein yield based on grain yield (A) and grain yield deviations (B); protein content (C) per se and protein deviations (D) as dependant variables in genomic-assisted (closed circles) forward predictions and for phenotypic selection with preliminary yield trial data (open circles)
